# Influence of rootstocks on fruit physical and chemical properties of peach cv. UFSun

**DOI:** 10.1002/fsn3.2005

**Published:** 2020-12-01

**Authors:** Shirin Shahkoomahally, Yuru Chang, Jeffrey K. Brecht, Jose X. Chaparro, Ali Sarkhosh

**Affiliations:** ^1^ Horticultural Sciences Department University of Florida Gainesville FL 32611 USA

**Keywords:** anthocyanin content, antioxidant capacity, flesh color, flesh firmness, phenolic content, *Prunus persica* L.

## Abstract

The subtropical peach cultivar UFSun grafted on five different rootstocks ('Flordaguard', 'Barton', 'MP‐29', 'P‐22', and 'Okinawa') was investigated in terms of the pomological and biochemical parameters of the fruit. Significant differences in fruit weight and size, soluble solids content, titratable acidity, and firmness were found among some rootstocks. The fruit length and diameter were different between the 'MP‐29' and other rootstocks. It was also found that firmness of 'UFSun' fruit was affected by the rootstock. The highest firmness value was found when 'UFSun' was grafted on 'Flordaguard'.' 'MP‐29' fruit had the highest soluble solids content, but there were no differences among the other rootstocks. 'UFSun' fruit from trees grafted on 'MP‐29' were smaller and had the most intense color compared to 'UFSun' fruit from other rootstocks. Rootstock had a significant influence on total fruit phenolic compounds, anthocyanin content, and total antioxidant activity, with fruit from 'UFSun' on 'MP‐29' having the highest values in all of them. A high correlation between fruit total antioxidant activity and total phenolic content was found. Overall, the results showed that 'MP‐29' seems to induce the highest fruit quality, showing higher contents of total soluble solids, total titratable acidity, total phenolic compounds, total antioxidant activity, and total anthocyanin content. Selecting the right combination of the rootstock and cultivar is important for optimizing fruit quality parameters.

## Graphical abstract

Significant differences in fruit weight and size, soluble solids content, titratable acidity, and firmness were found among some rootstocks. 'MP‐29'rootstock seems to induce the highest fruit quality, showing higher contents of total soluble solids, total titratable acidity, total phenolic compounds, total antioxidant activity, and total anthocyanin content. Selecting the right combination of the rootstock and cultivar is important for optimizing fruit quality parameters.

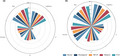

## 1 INTRODUCTION

Grafting is commonly used in many woody perennial crops to manipulate the scion phenotype indirectly, and appropriate rootstock selection is critical for any orchard (Reig et al., 2020). When rootstocks are selected for breeding, several aspects are taken into consideration such as vigor (Zarrouk et al., 2005), compatibility with the selected cultivar, adaptation to different soil types (Felipe, 2009; Pinochet, 2010) yield and water conditions to improve its use (Albás et al., 2004; Mestre et al., 2017; Reig et al., 2020; Tavarini et al., 2011; Yano et al., 2002), and resistance to biotic and abiotic stresses, including pathogens, diseases, and potentially toxic compounds in the soil such as salt or heavy metals, and training systems and improved fruit quality (Monet & Bassi, 2008; Rato et al., 2008).

In addition to adaptation properties, some authors have reported that rootstocks have a significant impact on fruit quality, including mineral composition and soluble solids content, organic acids content, and antioxidant content, such as reported in sweet cherry (Usenik et al., 2010) and peach (Albás et al., 2004; Font i Forcada et al., 2019; Giorgi et al., 2005; Iglesias et al., 2019; Mestre et al., 2017; Orazem et al., 2011; Reig et al., 2016; Remorini et al., 2008; Usenik et al., 2010). In a subsequent study, it was concluded that rootstocks with a higher plum genetic background (up to 50%) had better influence on fruit quality in term of phytochemical composition, organic acids, and sugars. Phenolic compounds in fruit play a significant role in several fruit quality traits, such as flavor, coloration, and health‐promoting and nutritional contents (Tomás‐Barberán et al., 2001). Drogoudi and Tsipouridis (Drogoudi & Tsipouridis, 2007) reported that rootstocks from three similar genetic origins had only minor effects on peach fruit antioxidant capacity. Significant differences among nine rootstocks in terms of peach fruit quality and nutritional characteristics, including total phenolics, antioxidant capacity, and ascorbic acid, were previously reported (Drogoudi & Tsipouridis, 2007). Scalzo et al. (Scalzo et al., 2005) found that the antioxidant capacity in fruit of 'Suncrest' peach grafted on different rootstocks was different. Tavarini et al (Tavarini et al., 2011) also reported different rootstocks in conjunction with drought stress influenced 'Suncrest' peach quality and nutritional properties. At present, little is known about the effect of rootstock on fruit phenolic composition.

Grafting on rootstocks with similar vigor but different genetic origins can result in different peach fruit quality, which indicates that vigor is not the only determinant affecting the scion (Giorgi et al., 2005; Orazem et al., 2011; Reig et al., 2016, 2020; Usenik et al., 2010). In several studies, the effect of rootstocks of diverse genetic origins on the physiology, tree vigor, yield, and fruit quality has been reported (Font i Forcada et al., 2012; Mestre et al., 2015). It was found that some vigorous rootstocks decreased fruit quality (Font i Forcada et al., 2012), while dwarfing rootstocks increased fruit quality by providing more nutrients as well as better light penetration into the canopy, consequently more efficient photosynthesis (Mestre et al., 2015). Additionally, a lack of chilling requirement in warm Tunisian conditions associated with the different origin and/or genetic background of several rootstocks for almonds and peaches resulted in bad adaptation to the growing conditions (Yahmed Ghrab et al., 2016; Yahmed et al., 2016. In temperate regions, recently, there is a notable reduction in chilling accumulation (Luedeling et al., 2009); therefore, it is expected that this reduction will be increased in the near future in warm Mediterranean areas (Ghrab et al., 2014; Luedeling et al., 2011). In fact, insufficient chilling accumulation significantly reduces peach production in a warm climate due to delayed and sporadic bud break (George & Erez, 2000; Ghrab et al., 2014). That is why low‐chill peach cultivars with lower chilling hours for bud break have been released for subtropical regions like Florida (Robert & Wayne, 2002).

'MP‐29' is an interspecific hybrid developed by the U.S. Department of Agriculture, Agricultural Research Service (USDA‐ARS) at Byron, GA, from a cross made in 1994 between a natural plum hybrid 'Edible Sloe' and an advanced red‐leaved peach rootstock selection from the Byron program (Maquilan, 2017). Based on trials in the main production areas of middle Georgia and South Carolina, peach scions budded to 'MP‐29' have displayed vigor similar to that of 'Sharpe' rootstock, but with higher yields of larger fruit, which increases yield efficiency. 'MP‐29' has red leaves similar to 'Flordaguard', which simplifies the identification and removal of rootstock suckers. 'MP‐29' is resistant to *Armillaria* root rot and also displays good resistance to peach tree short life (PTSL), similar to that of 'Guardian' rootstock (Beckman et al., 2012). Also, 'Flordaguard', a red‐leaved peach rootstock, was released by the University of Florida in 1991 (Sherman et al., 1991). 'Flordaguard' is the predominant rootstock throughout the state orchards, especially in orchards where the root‐knot nematode, *Meloidogyne floridensis*, and other root‐knot nematodes are found (Sarkhosh, et al., 2018). 'Flordaguard' is used as a rootstock for the production of peach, nectarine, and plum in low‐chill environments and on nonalkaline soils. 'P‐22' is another peach seedling rootstock, which is a hybrid between 'Guardian' and 'Flordaguard' with resistance to *Armillaria* root rot and PTSL (Beckman, *pers comm*). 'Okinawa' rootstock was introduced by the Florida Agricultural Experiment station from the Ryukyu Islands in 1953. It has been a promising nematode resistant rootstock for peach. Also, it has a low chilling requirement, which makes it well adapted for peach production in Central Florida. Soon after its introduction in Florida, it was reported that 'Okinawa' had good stands in the nursery as well as good compatibility with several peach varieties and selections (Sharpe, 1957).

Total antioxidant capacity and phenolic compounds have a significant impact on fruit quality, aroma, and flavor. Phenolic compounds are also the primary source of natural antioxidants in peach fruit (Chang et al., 2000). It is known that antioxidants can improve the oxidative stability of low‐density lipoprotein (LDL) in humans and play an essential role in reducing human atherosclerosis and coronary heart disease (Steingerg, 1989). In addition, chlorogenic acid, which is a kind of phenolic compound, is closely related to pigmentation and browning reactions in peach periderm tissue (Cheng & Crisosto, 1995), and in resistance to brown rot (*Monilia fructicola*) disease (Bostock et al., 1999). The total antioxidant capacity measurement provides a critical basis for comparing fruit nutritional levels (Wang et al., 1996). However, there may be considerable variation in the peach antioxidant content among different genotypes (Chang et al., 2000; Gil et al., 2002; Mestre et al., 2015). Anthocyanins are important secondary metabolites and specifically serve roles in fruit coloration, pollinator attraction and pollen dispersion, pathogen resistance, and protection of the chloroplast apparatus. Additionally, anthocyanins have a role in human nutrition and show great health benefits in the prevention of chronic diseases, such as cardiovascular diseases and cancer (Zafra‐Stone et al., 2007).

Production of high‐quality fruit is an important consideration, not only for peach growers, but also for consumers looking to benefit from the fruit's health‐promoting effects; thus, it is necessary to obtain information on which scion–rootstock combinations would produce optimum fruit quality. This study aimed to determine the variability of physical and chemical properties of the subtropical 'UFSun' peach grafted on five different rootstocks.

## 2 MATERIALS AND METHODS

### 2.1 Plant materials and field plot

The experiment was carried out on 6‐year‐old 'UFSun' peach grafted on five rootstocks (Table 1, (Maquilan, 2017)) at the Plant Science Research and Education Unit of the University of Florida, located in Citra, Florida (29° 24′ 42.01″ N; −82° 06′ 36.00″ W) in 2018 and 2019. Monthly averages of minimum, medium, and maximum air temperatures (°C), air relative humidity (%), and monthly total rainfall (mm) were recorded by the Florida Automated Weather Network (FAWN) weather station located on‐site (Table 2). 'UFSun' peach is adapted to central and south‐central Florida, has a chilling requirement of 100 chilling hours, and a fruit development period (FDP) from bloom to harvest of approximately 80 days when budded on 'Flordaguard' rootstock (Rouse & Sherman, 2003). 'UFSun' is a nonmelting‐flesh peach cultivar released in 2004 (Rouse et al., 2004). 'UFSun' trees bear heavy annual crops of early‐season, medium‐sized fruit, with yellow flesh and clingstone pits. 'UFSun' fruit are uniformly symmetrical and develop 50%–60% red skin with darker red stripes. When grown in south‐central Florida, fruit ripen in early April and 'UFSun' is one of the first commercial peaches to ripen in North America (Sarkhosh, et al., 2018). Soil in the trial site was classified as Entisol (USDA soil classification, (Mylavarapu et al., 2016). Peach trees were planted at a spacing of 6 m between and 4 m within rows and trained in an open‐vase system to prevent vigor interferences between adjacent trees. N‐P‐K fertilization (Super Rainbow 10‐10‐10, by Agrium), 1.36 kg (3 1bs) per application per tree, was applied three times per season. Summer pruning was conducted shortly after harvest, and winter pruning was performed similarly as in a commercial orchard (Taylor, 2009). Fruit were thinned 35 days after full bloom (in mid‐March), leaving one fruit approximately per 15 cm on the long shoot and one fruit on the short shoot. An automated drip irrigation system was used for tree irrigation. The orchard was irrigated with a dose of 1.6 L/hr per day during the summer. The rootstocks 'MP‐29', 'P‐22', 'Flordaguard', 'Okinawa', and 'Barton' were evaluated in this study (Table 1). There were 10 trees of each rootstock planted in a randomized complete block design (RCBD) with six block replicates. Fruit from the trees on different rootstocks were simultaneously harvested at approximately 80 days after full bloom (in second and third week of April) at the same maturity (ripeness stage, when the fruit ground color was more yellow than green) two times, 1 week apart. The sample consisted of 20 fruit from each tree, 5 fruit from each quadrant (N, S, E, W). In total, 480 fruit per treatment/combination (480 × 5 = 2,400 fruits) were harvested for fruit quantitative and qualitative analyses.

**Table 1:** Characteristics and origins of the peach rootstocks tested (Maquilan, 2017)

Rootstock	Chilling requirement (cu)	Species	Origin	Growth habit & vigor	Nematode resistance	Reference
'Flordaguard'	150–300^a^	Complex parentage (*P. persica* and *P. davidiana*)	Released by the University of Florida in 1991	Long limbs with whippy growth	Combined root‐knot nematode resistance from its 'Okinawa' ancestor with resistance to *Meloidogyne floridensis*	Sherman et al. (1991)
'Okinawa'	150^a^	Peach seedling line introduced as seed	Florida Agricultural Experiment Station (originally from Ryukyu, Japan in 1953)	Upright, good vigor	Resistant to *M. incognita*, *M. arenaria*, and some isolates of *M. javanica*; susceptible to *M. floridensis*	Sharpe et al. (1969); Sharpe, (1957)
'Barton'	400^a^	Coastal rootstock derived from a feral peach seedling	Northern NSW Australia	Upright, good vigor	Showed good root‐knot nematode resistance in the field	J. X. Chaparro (*pers. comm*.)
'P−22'	500^b^	Hybrid peach seedling line	USDA‐ARS rootstock breeding program, USA	Upright, good vigor	Performed similarly to 'Flordaguard' on field soils in Georgia and Florida infested with *M. incognita* and *M. floridensis*, respectively	T. G Beckman (*pers. comm*.)
'MP−29'	750^c^	Clonal plum × peach interspecific hybrid	Released for grower trial in 2011 by USDA‐ARS (Byron, Georgia) and Florida Agricultural Experiment Station	Semispreading, moderate vigor	Appeared to be resistant to *M. incognita* and *M. floridensis* in field trials in Georgia and Florida, respectively; resistant to *Armillaria* root rot and peach tree short life	Beckman et al. (2012)

^a^ Based on bloom time with standard reference cultivars at Gainesville, FL.

^b^ Based on bloom time with standard reference cultivars at Byron, GA.

^c^ Vegetative budbreak coinciding with peach cultivars requiring ≈750 hr of chilling below 7°C at Byron, GA.

**Table 2:** Monthly total rainfall, mean, maximum, and minimum temperatures, relative humidity, and chilling hours (0°C–7.2°C) in 2018 and 2019, in Citra, Florida, US

Month	Rainfall (mm)	Average maximum temp (°C) at 2 meters	Average minimum temp (°C) at 2 meters	Average Temp (°C) at 2 meters	Average air relative humidity (%)	Chill hours (<7°C)
August 2018	195.3	32.3	23.2	26.8	86.7	0
September 2018	266.1	30.9	21.1	25.5	83.4	0
October 2018	54.8	28	17.7	22.3	80.7	20
November 2018	78.4	24.8	12	17.8	84.1	5.5
December 2018	40.6	21.1	9.2	14.6	84.1	83
January 2019	132.8	17.2	4.8	10.6	77.8	204
February 2019	63.7	25.7	14	19.5	83.5	13.5
March 2019	80.2	23	8.4	15.6	67.9	0
April 2019	170.6	26.3	14.1	19.9	77.2	0
May 2019	205.4	29.1	18.3	23.5	81.2	0
June 2019	85.6	31.9	22.2	26.1	84.6	0
July 2019	165.3	31.5	22.6	26.1	87.8	0

### 2.2 Fruit physical properties

The fruit weight was measured with a Sartorius digital balance (±0.01 g sensitivity, Sartorius AG, Göttingen, Germany). Fruit length and diameter were measured with a digital caliper. Fruit shape was determined by calculating the ratio of fruit length to diameter. In addition, fruit dry matter content (%) was also measured after drying tissue samples consisting of epicarp and mesocarp at 45°C until a stable weight was achieved ( Lopez et al., 2010).

#### 2.2.1 Color measurement

Fruit peel and flesh color coordinates (CIE L*, a*, b*) were measured with a Minolta CR‐400 colorimeter (Konica Minolta) (Fundo et al., 2019). It was calibrated with a white blank calibration tile before each measurement. For peach peel color, the reddest and greenest parts of each fruit were measured. For peach flesh color, a peeler was used to remove the peel to a depth of 2 mm on the same areas of each cheek and the exposed surface was measured. Luminance coordinate L * means the whiteness value of the color from 0 (black) to 100 (white). The chromaticity value a* corresponds to red when positive and green when negative. The chromaticity coordinate b* corresponds to yellow when positive and blue when negative (Fundo et al., 2019). The hue and chroma were calculated from a* and b* values, with hue calculated as tan^−1^ (b*/a*) and chroma as [a* + b*]^1/2^.

#### 2.2.2 Fruit firmness

Flesh firmness was measured using a texture analyzer (Texture Technologies) equipped with an 8‐mm diameter convex tip, Magness–Taylor type probe (Gomez et al., 2005). The measurement was conducted at a pretest speed of 1 mm/s until fruit contact, test speed of 2 mm/s, and distance of 8 mm using a 50 kg calibrated load cell. The probe was inserted in the middle of the reddest and greenest parts of the cheek areas of each fruit, where the fruit had been peeled for flesh color measurement. Firmness values were recorded as the mean peak compression force and expressed in Newtons.

### 2.3 Chemical properties

#### 2.3.1 Total soluble solids (TSS), titratable acidity (TA), TSS/TA ratio, pH, and electrical conductivity (EC)

Fruit peel and flesh samples were homogenized using a blender and centrifuged for 20 min at 7,741*g* at 4°C to separate the juice from the insoluble cell material. Total soluble solids (TSS) and titratable acidity (TA) measurements were carried out on the fruit juice collected after centrifuging. The TSS was determined with an automatic refractometer (Reichert R2i300), the results being expressed as a percentage of TSS (at 20°C). The TA was measured using an automatic titrator (Metrohm 814 USB Sample Processor, Herisau, Switzerland). The juice sample was titrated to pH 8.2 with 0.1 M NaOH after recording the initial pH and was expressed as a percentage of malic acid equivalents in juice. The TSS/TA ratio was also calculated based on the TSS and TA values. The electrical conductivity (EC) of the juice was measured with an EC meter (Thermo Fisher Scientific) and was expressed as ms/cm (Pankaj et al., 2017).

#### 2.3.2 Determination of total antioxidant activity (TAA)

The ferric reduction capacity of the fruit tissues was determined using the ferric reducing antioxidant power (FRAP) assay (Benzie & Strain, 1996). The FRAP reagent was made from 300 mmol/L acetate buffer (pH 3.6) and 10 mmol 2,4,6‐Tris(2‐pyridyl)‐1,3,5‐triazine (TPTZ) in a 40 mmol/L HCl solution with 20 mmol/L FeCl_3_ in 10:1:1 ratio. In a yellow light environment, 100 μl of the samples and 100 μl of the different concentrations of standard solutions (Trolox) were mixed with 900 μL FRAP reagent separately. Then, the samples and the standard were read at 595 nm with a microplate reader (Synergy HTX Multi‐Mode ReaderBiTek). The standard Trolox curve was made with a concentration range of 10–800 μmol Trolox equivalents (TE). The TAA was expressed as μmol TE m/l.

#### 2.3.3 Determination of total phenolic content (TPC)

Total phenolic content (TPC) was determined by the modified colorimetric Folin–Ciocalteu method (Singleton & Rossi, 1965). A mixture of fruit peel and flesh was extracted with a mixture of 30 ml formic acid, 600 ml methanol, and 370 ml water and kept in a refrigerator overnight at 4°C. A 300 µl aliquot of extract supernatant was mixed with 300 µl Folin–Ciocalteu reagent and 300 µl sodium carbonate. The mixture was left in a dark room to stand for 60 min at room temperature before measuring the absorbance at 760 nm with a Biotech microplate reader (Synergy HTX Multi‐Mode Reader, BioTek). The same procedure was applied for standard solutions of different concentrations of gallic acid. The phenolic content was expressed as gallic acid equivalents (GAE).

#### 2.3.4 Total anthocyanin content (TAC)

Total anthocyanin content was measured according to the pH‐differential method (Giusti & Wrolstad, 2001) by two buffer systems: 0.4 M sodium acetate (pH = 4.5) and 0.025 M potassium chloride (pH = 1). A mixture of fruit peel and flesh was extracted with a mixture of 30 ml formic acid, 600 ml methanol, and 370 ml water and kept in a refrigerator overnight at 4°C. After centrifuging, 600 µl supernatant was mixed with 2.4 ml of the sodium acetate and potassium chloride buffers. The absorbance of the samples was measured at 510 nm and 700 nm using a Biotech microplate reader. The measurements of each peach sample were made in triplicate (*n* = 3) at room temperature (~22°C). Anthocyanin content was expressed as equivalents of cyanidin‐3‐glucoside (C‐3‐G) calculated using the following equation:

$$A={\left(A_{\text{pH}1.0}‐A_{\text{pH}4.5}\right)}_{525\;\text{nm}}‐{\left(A_{\text{pH}1.0}‐A_{\text{pH}4.5}\right)}_{700\;\text{nm}}$$

C‐3‐G(mg/L)=(A×MW×DF×1000)/L×ε

where A = absorbance; TA = total anthocyanin content (mg/L); MW = molecular weight; DF = dilution factor; l = pathlength; ε = molar extinction coefficient; and 1,000 = conversion factor from gram to milligram.

### 2.4 Statistical analysis

The data obtained from this study were analyzed with SAS version 11.0. One‐way ANOVA was analyzed with different rootstock genotypes as the main effect and replication as a random effect in a completely randomized plot analysis to determine the differences among rootstocks in physical and chemical fruit properties. Means were separated using Duncan's multiple range test at a significance level of *p* < .05. Pearson correlation matrix method was used to study correlations among all rootstocks for the fruit physical and chemical properties evaluated. A principal component analysis (PCA) was performed to assess the interrelationships among genotypes. Moreover, rootstock means were used in multivariate analysis to generate two‐way similarity cluster diagrams based on rootstocks similarity and variation.

## 3 RESULTS AND DISCUSSION

### 3.1 Fruit physical attributes

Fruit weight differed significantly between 'MP‐29' and the other rootstocks (Table 3). The lightest fruit were harvested from 'MP‐29' (104 g), while 'Flordaguard' and 'Okinawa' produced the heaviest fruit on average (128 g). This information is useful in the weight and dimension grouping in terms of the calculation and design of optimal packaging (Khanali et al., 2007; Shahbazi & Rahmati, 2013).

**Table 3:** Rootstock effect on physical properties of 'UFSun' peach grown in Florida

Rootstock	Branch length (cm)	Leaf area (cm^2^)	Fruit weight (g)	Fruit length, L (mm)	Fruit diameter, D (mm)	Fruit shape (L/D)	Dry matter (%)	Firmness (N)
'Barton'	60.1 a	40.2 a	123 a	62.1 a	61.7 ab	1.01 a	0.13 b	19.3 bc
'Flordaguard'	52.4 ab	38 ab	128.3 a	63.2 a	62.4 a	1.01 a	0.13 b	26.6 a
'MP‐29'	48.2 b	29.2 c	103.5 b	59.4 b	57.5 b	1.03 a	0.14 a	21.7 ab
'Okinawa'	59.9 a	37.8 ab	128 a	63.2 a	64.7 a	0.98 a	0.13 b	14.2 c
'P‐22'	56.5 a	35.4 b	122.9 a	62.6 a	61.8 a	1.01 a	0.13 b	15.1 c

Note

Different letters within columns indicate significant differences among rootstocks to Duncan multiple range test (*p* < .05).

The fruit with the smallest dimensions were produced by the rootstock 'MP‐29' (Table 3). Despite lower values of 'MP‐29' for fruit length and diameter, all rootstocks produced fruit of statistically similar shape. The shape of fruit from 'Barton', 'Flordaguard', and 'P‐22' was close to spherical (fruit shape = 1.01), while fruit from 'MP‐29' were more elongated (1.03) (Table 3). Drogoudi and Tsipouridis (Drogoudi & Tsipouridis, 2007) reported similar results for the effect of various rootstocks on fruit shape. 'MP‐29' rootstock produced fruit with the highest dry matter, but dry matter was not significantly different among the rest of the studied rootstocks (Table 3).

The highest firmness values were found in 'UFSun' fruit grafted on 'Flordaguard' with an average of 26.7 N and on 'MP‐29' at 21.69 N (Table 3), whereas the lowest were from 'Okinawa' (14.2) and 'P‐22' (15.1). The elevated firmness values induced by 'Flordaguard' and 'MP‐29' might indicate that fruit produced on these rootstocks can stay longer on supermarket shelves when fruit are harvested at the same maturity.

**FIGURE 1:**
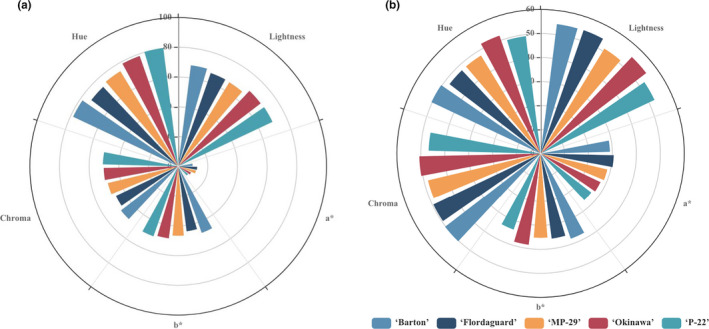
Color parameters of fruit peel (a) and flesh (b) of 'UFSun' peach grown on five peach rootstocks in Florida

The lightest colored flesh and peel (high L value) was found in fruit from 'Okinawa' and 'Barton', whereas the darkest colored flesh was found in fruit from 'MP‐29' (Figure 1). Fruit produced on 'Flordaguard' had the reddest peel and flesh (high a^*^ values), whereas fruit from 'Okinawa' had the most yellow flesh and peel (high b^*^ values). Fruit from 'Okinawa' showed the highest hue and chroma values in the fruit flesh, indicating that those fruit were more yellow than orange. In contrast, 'Flordaguard' and 'MP‐29' had the lowest average peel and flesh hue values, indicating that they were more orange. Chroma value describes color purity, while the hue angle represents the shade of color as a coordinate in a standardized color space.

### 3.2 Fruit chemical attributes

Higher TSS values were obtained from fruit on 'MP‐29' compared with the other rootstocks (Table 4) confirming previous observations by Maquilan (Maquilan, 2017) that TSS content corresponded with fruit size and is independent of the rootstock. This is also consistent with other findings that TSS is not strongly affected by the rootstock (Giorgi et al., 2005; Orazem et al., 2011). However, other studies reported differences in TSS (Mestre et al., 2017; Reig et al., 2016, 2020). Therefore, depending on the cultivar, rootstock, years of evaluation, soil conditions, and climate, TSS could be more or less affected by rootstock.

**Table 4:** Chemical attributes of 'UFSun' fruit produced on different rootstocks

Rootstock	TSS (%)	TA (%)	TSS/TA ratio	pH	EC (ms/cm)	TAA (mmol Te/L)	TAC (mg/100 g)	TPC (mg GA/L)
'Barton'	11.70 b	0.48 ab	24.18 a	3.89 a	3.89 a	0.81 c	4.27 b	571.83 c
'Flordaguard'	11.76 b	0.47 b	25.05 a	3.93 a	3.97 a	1.03 b	5.47 b	620.13 bc
'MP‐29'	13.10 a	0.54 a	24.11 a	3.74 b	3.58 b	1.60 a	15.03 a	749.84 a
'Okinawa'	11.52 b	0.45 b	25.41 a	3.95 a	3.87 a	0.80 b	5.17 b	557.54 c
'P‐22'	11.81 b	0.50 ab	23.62 a	3.87 a	3.81 a	1.09 c	7.21 b	649.24 b

Note

Different letters within columns indicate significant differences among rootstocks by Duncan's multiple range test (*p* < .05).

Abbreviations: EC, electrical conductivity; TA, titratable acidity; TSS, total soluble solids, TAA, total antioxidant activity; TAC, total anthocyanin content; TPC, total phenolic compounds.

'Okinawa' showed a tendency to attain the lowest TA values with an average of 0.45%. 'MP‐29' had the highest average TA with 0.54% (Table 4). Similarly, it was previously found that some rootstocks induced higher acidities than other rootstocks (Mestre et al., 2017; Ortega et al., 2013; Reig et al., 2016, 2020). Statistically, there were no significant differences in the ripeness index (RI = TSS/TA) for the studied rootstocks (Table 4). Significant differences in the RI have also not been found in other studies (Ortega et al., 2013). However, 'Okinawa' tended to induce a higher RI with an average of 25.41. By contrast, 'P‐22' had the lowest average RI at 23.62. Concerning pH and EC, significant differences between 'MP‐29' and other rootstocks were found. 'MP‐29' fruit had significantly lower pH and EC values than those of 'Okinawa', 'Barton', 'P‐22', and 'Flordaguard' (Table 4). However, there were no significant differences among the other rootstocks. This is in agreement with other studies on cherry, showing that there were no rootstock effects on the pH of cherry juice (Cantín et al., 2010; Gonçalves et al., 2006; Jiménez et al., 2004). Poor physical properties, higher TSS and TA, and lower pH and EC were observed in fruit from 'MP‐29'. One possible explanation is that fruit TSS and TA of 'MP‐29' were concentrated due to the smaller fruit size and lower juice yield (higher dry matter content). It is reported that 'MP‐29' is a semispreading rootstock with moderate vigor (Beckman et al., 2012), which is reflected by the shorter branch length and smaller leaf area obtained from the present study. Although 'MP‐29' is a hybrid between plum and peach, no graft incompatibility has been reported with 'MP‐29' as rootstock in peaches and none was observed in the present study. Beckman, Chaparro (Beckman et al., 2012) also reported that 'MP‐29' had lower cumulative mortality over 'Sharpe' and 'Guardian' in two sites in Georgia for a period of 10 years. Another possibility is that the accumulated chilling units (CU) in the location of our study are insufficient for 'MP‐29', because 'MP‐29' requires 750 cu, but mid‐Florida can only reach 400 cu. Insufficient chilling in regions with warm winters can lead to deformation of buds, delayed bud break and bloom date, and partial or uneven vegetative and flower buds germination, resulting in low fruit set and yield (Kwon et al., 2020). A similar experiment in a higher chill area is needed to evaluate 'MP‐29's performance with enough chilling units.

**FIGURE 2:**
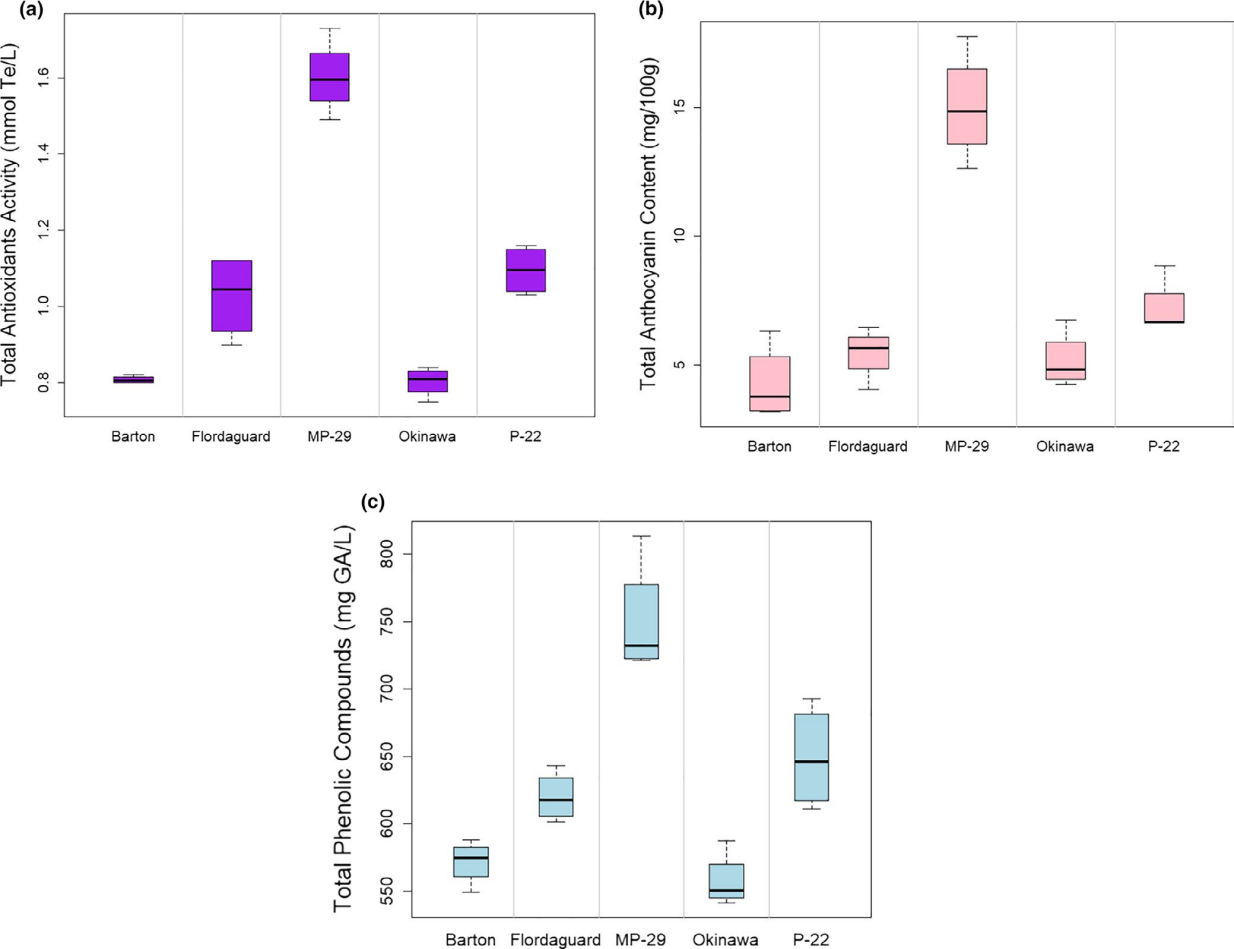
Box plot of total antioxidant activity (a), total anthocyanin content (b), and total phenolic compounds (c) in fruit of 'UFSun' peach grown on various peach rootstocks

The highest TAA was found in fruit from 'MP‐29', followed in descending order by 'P‐22', 'Flordaguard', 'Barton', and 'Okinawa' (Table 4). 'MP‐29' fruit also had higher TAC than the other rootstocks (Table 4). These results were similar to other findings for stone fruits and other tree fruits (Chang et al., 2000; Font i Forcada et al., 2013; Gil et al., 2002; Giorgi et al., 2005; Reig et al., 2016; Scalzo et al., 2005). Drogoudi and Tsipouridis (Drogoudi & Tsipouridis, 2007) suggested that the rootstock slightly influenced clingstone peach fruit antioxidant content and other chemical characteristics due to close genetic origin among studied rootstocks. The higher TAA, TAC, and TPC on 'MP‐29' compared with the other rootstocks could be due to the lower fruit weight having a concentrating effect on the fruit chemical composition of 'UFSun'. 'MP‐29' has a large plum species component in it. The other four rootstocks each have a large peach species component in them. Therefore, this may explain the differences between 'MP‐29' and the other rootstocks (Figure 2). Furthermore, 'MP‐29' is more dwarfing to peach than the other rootstocks, so that could be a factor in better providing more nutrients to the scion and light penetration into the canopy, consequently more efficient photosynthesis impacting fruit quality with this cultivar (Font i Forcada et al., 2012; Mestre et al., 2015).

### 3.3 Multivariate analysis

To investigate the relationship among physical and chemical properties, Pearson's correlation coefficient was calculated (Table 5). A positive linear relationship was found between the fruit length and width (*r* = .75). There was a high correlation between fruit weight and fruit length (*r* = .97), and fruit diameter (*r* = .76). Also, there was a strong negative correlation between dry matter and fruit weight (*r* = −.83, *p* < .05) (Table 5). A negative correlation was found between TSS and fruit weight (*r* = −.82) (Table 5), with the highest TSS value corresponding to 'MP‐29' with the lowest fruit weight. Furthermore, a positive correlation between TA and TSS (*r* = .58) was found. There was a negative correlation between RI and TA (*r* = −.72). Total anthocyanin content and TPC positively correlated with TAA (*r* = .88 and *r* = .96, respectively), indicating the great contribution of phenolic compounds to TAA. Gil, Tomás‐Barberán (Gil et al., 2002) reported that the antioxidant mechanism in plant tissues is more closely related to hydrolyzable tannins than to anthocyanin content. However, Reig, Mestre (Reig et al., 2016) reported a moderate correlation between TAA and TPC, but no correlation between TAA and total anthocyanins content when 'Big Top' nectarine was grafted on peach‐ and plum‐based rootstocks under Mediterranean climatic conditions.

**Table 5:** Correlation matrix of physicochemical and biochemical properties

	1	2	3	4	5	6	7	8	9	10	11	12	13	14	15	16	17	18	19
1. Fruit weight	1.00																		
2. Fruit diameter	**0.76**	1.00																	
3. Fruit length	**0.97**	**0.75**	1.00																
4. Fruit shape (L/D)	−0.43	**−0.89**	−0.38	1.00															
5. Dry matter	**−0.83**	**−0.60**	**−0.85**	NS	1.00														
6. Branch length	0.49	**0.60**	**0.51**	**−0.52**	**−0.52**	1.00													
7. Chroma (Peel)	NS	NS	NS	NS	NS	NS	1.00												
8. Hue (Peel)	NS	NS	NS	NS	NS	NS	NS	1.00											
9. Chroma (Flesh)	NS	NS	NS	NS	NS	NS	NS	NS	1.00										
10. Hue (Flesh)	NS	NS	NS	−0.45	NS	0.39	NS	0.47	−0.49	1.00									
11. TAA	**−0.84**	**−0.70**	**−0.79**	0.47	**0.77**	**−0.73**	NS	NS	NS	NS	1.00								
12. TAC	**−0.89**	**−0.72**	**−0.81**	0.48	**0.77**	**−0.61**	NS	NS	NS	NS	**0.88**	1.00							
13.TPC	**−0.78**	**−0.70**	**−0.74**	**0.50**	**0.73**	**−0.72**	−0.39	NS	NS	NS	**0.96**	**0.80**	1.00						
14. Firmness	NS	NS	NS	NS	NS	−0.47	NS	NS	−0.46	**−0.53**	NS	NS	NS	1.00					
15. TSS	**−0.82**	**−0.66**	**−0.83**	NS	**0.89**	**−0.69**	NS	−0.38	NS	NS	**0.85**	**0.75**	**0.82**	NS	1.00				
16. pH	**0.87**	**0.61**	**0.79**	NS	**−0.74**	NS	0.39	NS	NS	NS	**−0.78**	**−0.82**	**−0.70**	NS	**−0.69**	1.00			
17. EC	**0.74**	**0.58**	**0.70**	NS	**−0.8**	0.38	NS	NS	NS	NS	**−0.72**	**−0.81**	**−0.69**	NS	**−0.70**	**0.82**	1.00		
18. TA	**−0.83**	**−0.65**	**−0.77**	0.39	**0.63**	NS	**−0.53**	NS	NS	NS	**0.71**	**0.75**	**0.68**	NS	**0.58**	**−0.89**	**−0.66**	1.00	
19. TSS/TA	NS	NS	NS	NS	NS	NS	0.47	NS	NS	NS	NS	NS	NS	NS	NS	**0.50**	NS	**−0.72**	1.00

Note

Absolute linear correlation coefficients *p* ≥ |0.50| are marked in bold. Results are not significant (NS) or significant *at* *p* < .05.

**FIGURE 3:**
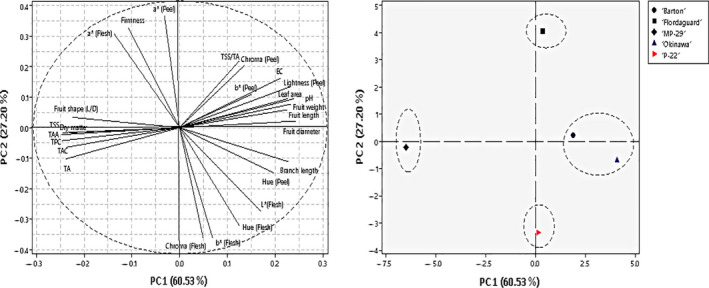
Principal components analysis of the first two factors showing the dispersion of rootstocks based on the measured parameters: variable plot (left) and observation score (right)

In order to determine rootstocks genotype dispersion, principal component analysis (PCA) was performed (Figure 3). There were eigenvalues for the first two components of 60.5 and 27.2, respectively, with 87.7% cumulative eigenvalues of data variance. Eigenvalues of the third and fourth PCA factors were negligible (8% and 3%), and therefore, they are not discussed further. The strongest negative correlations in PC1 were with TAA, TAC, TPC, TA, and TSS, while fruit diameter, branch length, and chromatic parameters had the strongest positive correlations. PC2 was strongly positively correlated with firmness and fruit shape, while it was negatively correlated with fruit weight and length (Figure 3a). Based on the physical and chemical properties, and the bioactive components of fruit on the various rootstocks, there were four groups of genotypes: (a) 'Flordaguard', (b) 'MP‐29', (c) 'Okinawa' and 'Barton', and (d) 'P‐22'. 'MP‐29' seems to be quite different from all of the other rootstocks on the first axis, where TSS, TAA, TPC, TA, and TAC have the strongest contributions, creating a distinct group on the negative side along the PC1. 'MP‐29' is characterized by significantly higher TPC, TAC, and TPC compared with the other studied rootstocks (Figure 3).

**FIGURE 4:**
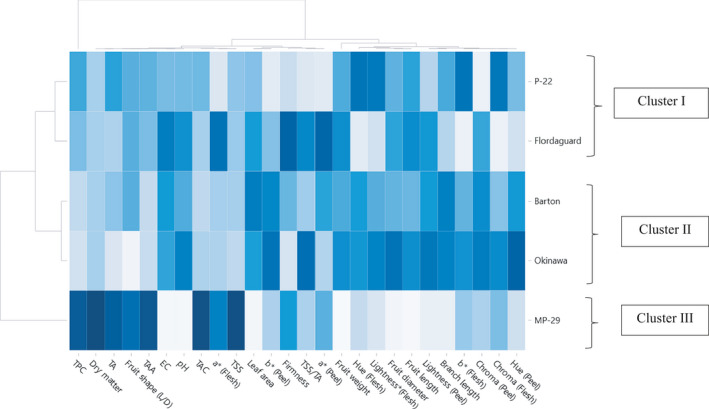
Cluster display of the various rootstocks grouped by similarities in physical and chemical properties mean values (Gradient from low [light blue] to high [dark blue])

Also, in order to group the rootstocks based on increasing dissimilarity, a hierarchical agglomerative cluster assessment was performed (Figure 4). The first group (Cluster I, Figure 4), which included the rootstocks 'Flordaguard' and 'P‐22', was characterized by fruit with medium to low dry matter and TSS, and medium to high TAA, EC, pH, fruit diameter, and fruit length. The second cluster (Cluster II, Figure 4), which included the rootstocks 'Okinawa' and 'Barton', showed medium to high color parameters for peel and flesh, fruit weight, fruit length, and fruit diameter values and medium to low values for TPC, dry matter, TA, TAA, TAC, and TSS. Within this cluster, 'Okinawa' had one of the highest color parameter values. The genotype included in the third group (Cluster III, Figure 4), 'MP‐29', showed the highest TSS, TAC, TAA, TA, TPC, and the lowest fruit length, diameter, pH, EC, and leaf area values.

## 4 CONCLUSION

In conclusion, selecting the proper rootstock is essential for obtaining the desired physical and chemical characteristics of peach fruit. Our results showed that fruit weight, size, firmness, TSS, TAA, and TAC were significantly affected by the choice of rootstock. Generally, the 'MP‐29' rootstock produced the best fruit quality (highest TSS, TA, TAA, TPC, and TAC). However, the 'MP‐29' rootstock also produced the smallest fruit and tree size. There is no doubt that the influence of the rootstock on fruit trees is much more complicated than some variables can measure. Results from the present study have demonstrated the fruit quality characteristics in widely grown and new peach rootstock selections.

## CONFLICT OF INTEREST

The authors declare no conflict of interest.

## ETHICAL APPROVAL

Ethics approval was not required for this research.

## Data Availability

The data that support the findings of this study are available from the corresponding author upon reasonable request.
